# A Comprehensive Review of Slag-Coating Mechanisms in Blast-Furnace Staves: Furnace Profile Optimization and Material-Structure Design

**DOI:** 10.3390/ma18163727

**Published:** 2025-08-08

**Authors:** Qunwei Zhang, Hongwei Xing, Aimin Yang, Jie Li, Yang Han

**Affiliations:** 1 College of Metallurgy and Energy, North China University of Science and Technology, Tangshan 063210, China; zhangqunwei@stu.ncst.edu.cn (Q.Z.); hwxing@ncst.edu.cn (H.X.); aimin@ncst.edu.cn (A.Y.); lijie@ncst.edu.cn (J.L.); 2 Tangshan Key Laboratory of Engineering Computing, North China University of Science and Technology, Tangshan 063210, China

**Keywords:** blast-furnace staves, slag-coating mechanisms, fractional-order heat-transfer models, furnace profile optimization, self-protection capability, multiphase coupling

## Abstract

Blast-furnace staves serve as critical protective components in ironmaking, requiring synergistic optimization of slag-coating behavior and self-protection capability to extend furnace lifespan and reduce energy consumption. Traditional integer-order heat transfer models, constrained by assumptions of homogeneous materials and instantaneous heat conduction, fail to accurately capture the cross-scale thermal memory effects and non-local diffusion characteristics in multiphase heterogeneous blast-furnace systems, leading to substantial inaccuracies in predicting dynamic slag-layer evolution. This review synthesizes recent advancements across three interlinked dimensions: first, analyzing design principles of zonal staves and how refractory material properties influence slag-layer formation, proposing a “high thermal conductivity–low thermal expansion” material matching strategy to mitigate thermal stress cracks through optimized synergy; second, developing a mechanistic model by introducing the Caputo fractional derivative to construct a non-Fourier heat-transfer framework (i.e., a heat-transfer model that accounts for thermal memory effects and non-local diffusion, beyond the instantaneous heat conduction assumption of Fourier’s law), which effectively describes fractal heat flow in micro-porous structures and interfacial thermal relaxation, addressing limitations of conventional models; and finally, integrating industrial case studies to validate the improved prediction accuracy of the fractional-order model and exploring collaborative optimization of cooling intensity and slag-layer thickness, with prospects for multiscale interfacial regulation technologies in long-life, low-carbon stave designs.

## 1. Introduction

### 1.1. Research Background and Industrial Needs

As the core equipment in the steel industry, blast furnaces play a pivotal role in achieving global “dual-carbon” goals through lifespan extension and efficiency enhancement [1]. Staves, the critical protective structures of furnace linings, balance thermal loads, reduce refractory erosion, and maintain stable furnace profiles through the dynamic formation and stabilization of slag coatings [2]. However, under unsteady smelting conditions, the complex multiscale coupling between the dynamic behavior of slag layers (e.g., periodic shedding and regrowth) and the thermomechanical response of staves directly impacts the furnace’s self-protection capability and service life [3]. In recent years, with increasing smelting intensity and aggravated raw material fluctuations, the traditional experience-driven design paradigm for staves has become inadequate to meet the demands of furnace intelligentization and longevity. This has necessitated an in-depth mechanistic exploration of how slag-coating capacity regulates furnace profile evolution [4,5].

### 1.2. Limitations of Existing Models

Current models for heat transfer in blast-furnace staves mainly rely on integer-order differential equations (e.g., Fourier’s law), which assume materials are homogeneous and heat transfers instantly [6,7]. However, the stave system inherently constitutes a multiphase heterogeneous medium comprising metallic coolers, refractories, and amorphous slag coatings, exhibiting pronounced non-Fourier characteristics during heat transfer [8,9]. Two key issues arise: (1) “thermal memory effects”: Micro-pores and cracks in slag coatings create complex heat flow paths, so heat transfer depends not just on current conditions but also on past thermal states; and (2) “non-local diffusion”: At the interface between refractories and metals, heat transfer is delayed and influenced by regions far from the interface. These phenomena mean traditional models cannot accurately predict slag-layer thickness or thermal resistance, limiting the precision of stave design and furnace profile control [10,11].

### 1.3. Innovations and Research Objectives

To address these limitations, this study proposes three key advancements:

(1) Cross-scale description via fractional-order heat-transfer modeling: For the first time, the Caputo fractional derivative is introduced into heat-transfer modeling of blast-furnace staves to construct a conduction equation incorporating temporal memory effects and spatial nonlocality [12,13]. This “non-Fourier” framework (where heat transfer is not instantaneous and depends on historical thermal states) better captures complex heat flow in porous structures and slow heat transfer at interfaces.
(2) Multifactor coupling mechanism: A two-factor driven model integrating slag-coating capacity and environmental conditions is established to reveal nonlinear influences of stave surface topography, material thermal expansion matching, and cooling intensity on slag-layer stability. By coupling thermodynamic protection, chemical isolation, and mechanical buffering mechanisms, a synergistic optimization framework of “material–structure–process” is constructed.
(3) Industry-oriented application: Leveraging industrial blast-furnace case data, zonal stave design criteria are proposed to lay the theoretical foundation for developing digital twin systems in blast furnaces.

### 1.4. Paper Structure

**Figure 1:**
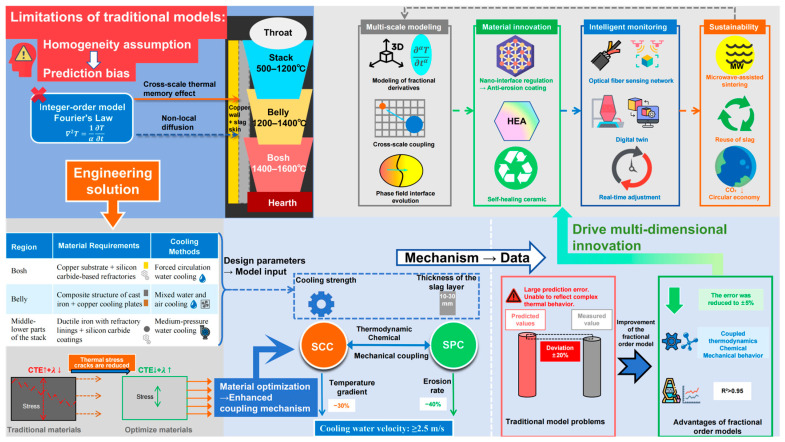
Overall framework diagram.

This paper is structured as follows: Section 2 discusses zone-specific stave designs in the bosh, belly, and lower stack, and how parameters like thermal conductivity affect slag coating; Section 3 establishes coupled thermodynamic, chemical, and mechanical models to clarify interactions between slag-layer evolution and stave damage resistance; Section 4 introduces fractional calculus to develop a heat-transfer model accounting for thermal memory and non-local diffusion; Section 5 identifies future challenges in multiscale modeling, new materials, intelligent monitoring, and low-carbon technologies; Section 6 summarizes key findings and prospects for achieving long stave life and carbon neutrality. The framework forms a complete system of “mechanism analysis–model innovation–engineering application,” providing theoretical support for optimizing blast-furnace cooling systems. The overall framework of the text is shown in Figure 1.

## 2. Regional Disparities in Structural and Material Design of Blast Furnace Staves

### 2.1. Structural Classification and Functional Adaptability

**Figure 2:**
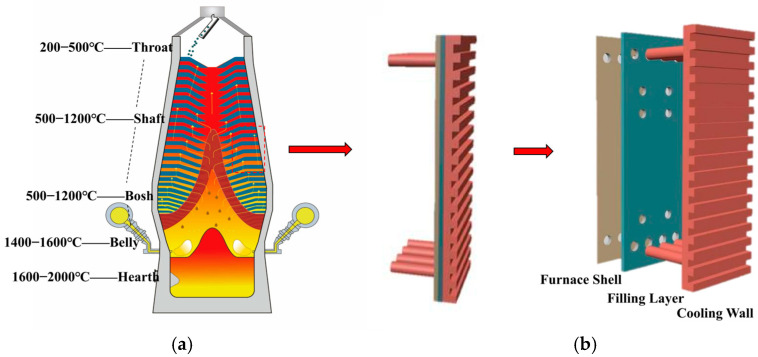
Comprehensive schematic diagram of blast-furnace structural zoning and cooling system: (**a**) blast furnace structural zoning; and (**b**) partial schematic of cooling stave structure.

The design of blast-furnace staves requires targeted optimization according to temperature gradients, thermal load intensity, and mechanical stress in different furnace zones, with the regional division and temperature distribution of the blast furnace shown in Figure 2a. The core objective is to maintain stable slag-coating capacity and self-protection mechanisms under extreme thermo-mechanical coupling environments. Blast-furnace stave designs are primarily categorized into the bosh, belly, and middle-lower parts of the stack regions [14,15,16], with the stave structure illustrated in Figure 2b.

The bosh is a high-temperature zone (1400–1600 °C) where smelting reactions are most intense. Staves here must withstand extreme heat and abrasion from materials and gas flow [17].

(1) Structure design: Copper staves with multi-channel cooling systems are used. Cooling water flow is adjusted dynamically to manage heat loads, and the high thermal conductivity of copper (≥380 W/(m·K)) promotes rapid slag solidification, forming a “copper wall + slag coating” double protective layer. The wall’s angle is optimized to help materials move downward smoothly, reducing slag detachment risks.
(2) Refractory parameters: Copper staves utilize high-purity copper substrates with surface-applied slag-erosion resistant coatings to enhance tolerance to molten slag and high-temperature gas flow. Silicon-carbide-based refractories are primarily employed as lining materials, balancing slag-erosion resistance and thermal-shock resistance.

The belly (1200–1400 °C) lies between the bosh and stack, where materials transition from solid to molten. It faces thermal shocks from hot metal and gas, plus mechanical loads from upper materials [18].

(1) Structure design: A combined system of copper cooling plates and cast-iron staves is adopted. Copper plates are embedded in cast iron to enhance cooling uniformity, especially at corners. The design avoids stress concentration to prevent slag-coating detachment from thermal expansion.
(2) Refractory parameters: Copper plates are reinforced with chromium and zirconium to improve thermal-shock resistance. Cast-iron staves use ductile iron (tensile strength ≥ 400 MPa) for mechanical durability. Lining materials include high-alumina bricks (Al_2_O_3_ ≥ 80%) or sialon-bonded corundum bricks, which resist alkali corrosion.

The middle-lower stack (500–1200 °C) sees materials transition from solid to softening, with erosion from high-temperature gases (CO, H_2_) and reduction reactions [19].

(1) Structure design: A hybrid cooling system is used: unlined copper staves in high-heat areas and ductile iron staves with refractory linings in medium-heat areas. Surface roughening (Ra = 10–50 μm) on staves helps slag adhere, and serpentine water pipes improve cooling uniformity. Transition refractories at stave-lining junctions reduce cracking from thermal expansion differences.
(2) Refractory parameters: Copper staves have anti-erosion coatings (e.g., Al_2_O_3_-SiC composites). Cast-iron linings use silicon-nitride–silicon-carbide bricks (porosity < 15%) to resist CO erosion and wear.

Based on the differences in thermal loads of various regions, the design of staves needs to be adjusted accordingly to ensure that the temperature remains within the design range, thereby effectively extending the service life of the furnace body [20]. For regions with high thermal loads, materials with high heat resistance, such as high-alumina bricks and refractory concrete, are recommended to cope with extreme working conditions; in regions with low thermal loads, materials with stronger abrasion resistance can be selected to improve their durability. The detailed design requirements for staves in different regions are presented in Table 1.

### 2.2. Influence of Refractory Parameters on Slag-Coating Capacity

Refractory properties directly affect slag-coating formation and stability [21,22]. Key parameters include thermal conductivity, surface roughness, and resistance to slag penetration.

(1) Thermal Conductivity and Thermal-shock Stability

**Figure 3:**
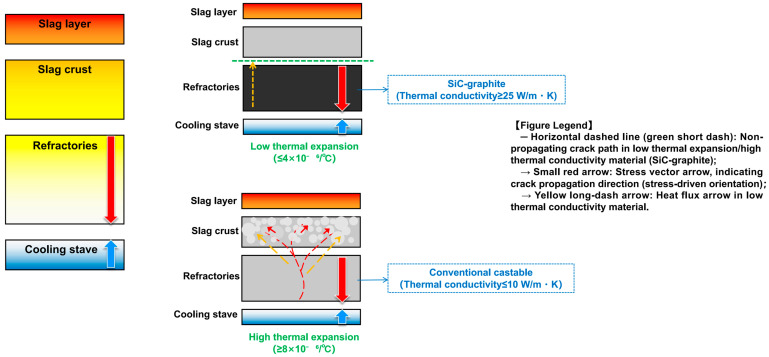
Schematic of thermodynamic mechanisms of refractory parameters on slag skin formation.

**Figure 4:**
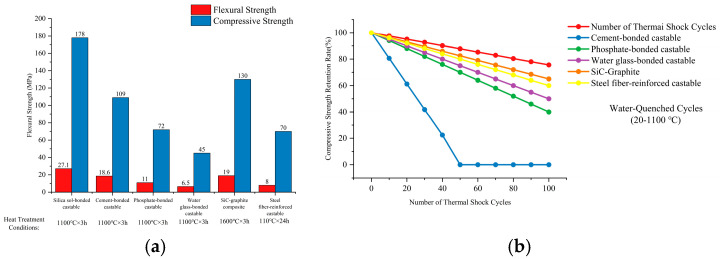
Comparison of mechanical properties and thermal-shock stability of castables with different bonding systems: (**a**) bar chart of mechanical properties; and (**b**) thermal shock stability curves.

The dynamic heat conduction and thermal-shock resistance of refractories work together to affect slag skin formation, with the mechanisms shown in Figure 3 and Figure 4.

Figure 3 (left) shows a cross-section of the blast furnace’s layered structure: the large red arrow indicates heat flowing naturally from high to low temperatures, while the large blue arrow represents the complete heat flow path (meaning “heat is eventually removed by the cooling system”); the main heat flow remains top-down.

Figure 3 (right) compares thermal conductivity and thermal expansion coefficient. In the upper right, high thermal conductivity materials (e.g., silicon–carbide–graphite composite castables, thermal conductivity ≥ 25 W/m·K) are shown with yellow dashed arrows, representing fast heat conduction. This quick heat dissipation shortens the time it takes for molten slag to solidify, helping form a uniform, dense glassy slag layer (light gray). Additionally, these composites have a low thermal expansion coefficient (≤4 × 10^−6^/°C), which stops thermal stress-induced microcracks from growing; their flexural strength retention rate after testing reaches 85% [23]. In contrast, the lower-right section shows that ordinary castables, with low thermal conductivity and high thermal expansion, result in porous slag skin and red branched cracks (long dashed lines), with only 40% flexural strength retained.

It should be noted that SiC–graphite composites exhibit anisotropic thermophysical properties due to the potential oriented arrangement of particles: the thermal conductivity parallel to the particle alignment direction can reach 25–30 W/(m·K), while that in the perpendicular direction drops to 10–15 W/(m·K); a similar difference is observed in thermal expansion coefficients (≤4 × 10^−6^/°C in the parallel direction, slightly higher in the perpendicular direction). This discrepancy leads to uneven temperature distribution on the stave surface, further affecting the uniformity of slag-layer solidification. In practical applications, the influence of anisotropy can be reduced by optimizing particle dispersion (e.g., random distribution).

Castables with different bonding systems differ in mechanical properties and thermal-shock stability, which further confirms the above coupling mechanisms. As shown in Figure 4a, after being heated at 1100 °C for 3 h, silica sol-bonded castables have room-temperature flexural strength of 27.1 MPa and compressive strength of 178 MPa—46% and 62% higher than cement-bonded castables (18.6 MPa and 109 MPa, respectively). In thermal-shock stability tests (Figure 4b), silica sol-bonded castables retain 75.6% of their compressive strength after 100 water quenching cycles (20–1100 °C), while cement-bonded ones develop through cracks after only 49 cycles [24].

This performance gap comes from the combined effect of their phase structure and heat conduction: silica-sol binders contain mullite phases with negative thermal expansion, which offsets the matrix’s deformation. Their moderate thermal conductivity also balances efficient slag skin formation and resistance to thermal stress damage. In contrast, cement binders have poorly matched high thermal conductivity and high expansion, which worsen structural instability during thermal shock and cause faster loss of mechanical properties.

Notably, excessively high thermal conductivity can lead to large temperature gradients in furnace walls. Thus, it must be paired with low thermal expansion (≤4 × 10^−6^/°C) to prevent thermal stress from causing microcracks. Performance data for other bonding systems (e.g., phosphate, water glass) in Figure 4 also support this pattern, providing quantitative references for selecting materials for blast-furnace staves [25,26].

(2) Surface Roughness and Wettability

A material’s surface texture and chemical properties together control how molten slag spreads and adheres. Rough surfaces (Ra = 10–50 μm) increase contact area with slag through mechanical anchoring, while tiny grooves draw slag into them via capillary action, creating 3D interlocked interfaces [27].

**Figure 5:**
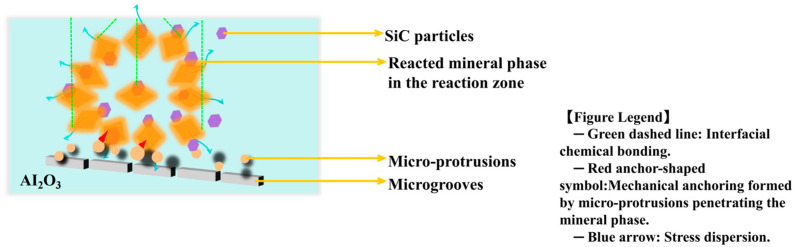
Schematic illustration of the interaction mechanism between rough surface and molten slag interface.

For example, as shown in Figure 5, Al_2_O_3_-SiC composite coatings work well at high temperatures due to their combined effects: the Al_2_O_3_ layer is chemically stable and stops slag from seeping in, while scattered SiC particles—with high thermal conductivity—improve resistance to thermal shock and reduce stress from temperature changes [28,29].

At the interface, high-melting minerals (such as anorthite) from the slag form physical locks with rough surface bumps. This “mechanical pinning” is marked by red anchor symbols in the figure. Meanwhile, green dashed lines at the Al_2_O_3_–slag interface represent chemical compatibility. These two mechanisms together make the interface bond stronger.

**Figure 6:**
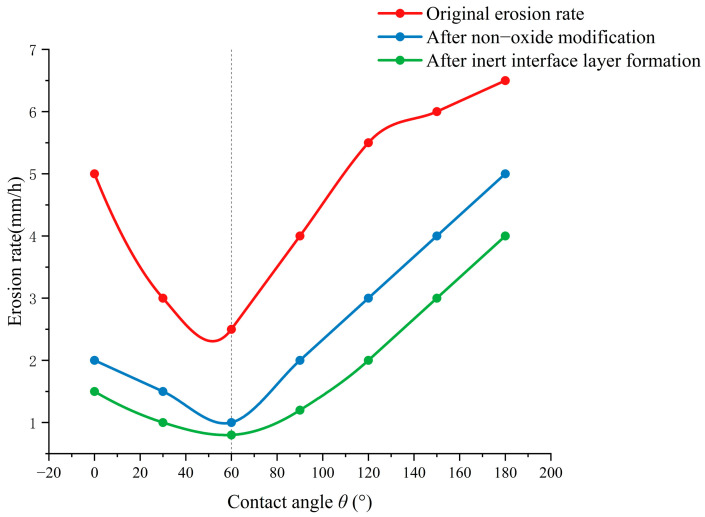
Wettability–erosion rate relationship curve.

Wettability optimization requires balancing interfacial thermodynamic equilibrium and reaction kinetics inhibition. As shown in Figure 6, low wetting angles (θ) generally facilitate continuous slag-layer coverage. However, when θ falls below the critical value of 60°, accelerated penetration of active components such as FeO and CaO leads to increased erosion rates [30]. Introducing non-oxide materials (e.g., Si_3_N_4_ or BN) to form gradient wetting layers can simultaneously reduce the θ angle (below 60°) and increase the interfacial energy barrier, thereby lowering erosion rates. Concurrently, in situ formation of inert interfacial layers such as spinel (MgAl_2_O_4_) or forsterite (Mg_2_SiO_4_) blocks direct reactions between molten slag and the substrate, further inhibiting erosion [31]. In Figure 6, the orange and yellow curves, respectively, show the inhibitory effects of non-oxide modification and inert interfacial layers on erosion rates, corroborating the synergistic optimization mechanism of “moderate wettability + interfacial energy barrier regulation.”

(3) Penetration Resistance and Wear Resistance

**Figure 7:**
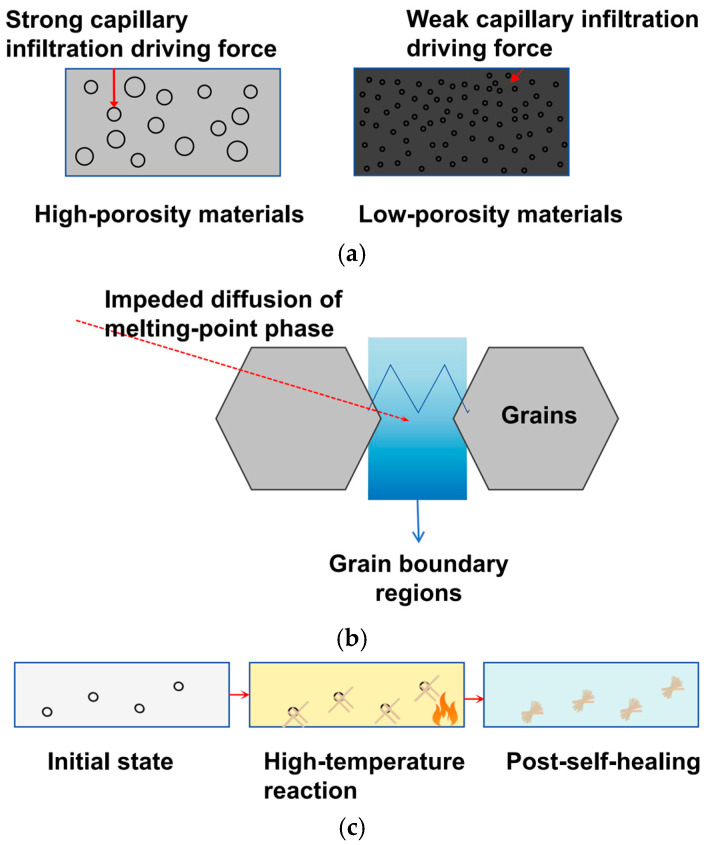
Penetration resistance mechanisms: (**a**) impact of apparent porosity and pore diameter; (**b**) diffusion hindrance by nanoscale grain; and (**c**) mullite self-healing mechanism.

Molten slag penetration depends heavily on a material’s microstructure—specifically its apparent porosity, pore size distribution, and grain boundary features—as shown in Figure 7a,b. When apparent porosity exceeds 15% and pores are larger than 1 μm (on the left in Figure 7a), slag easily seeps through connected pores because the capillary resistance is too weak. In contrast, when porosity is ≤15% (on the right in Figure 7a), the force driving slag into pores drops sharply as pore size shrinks, significantly reducing capillary suction of molten slag [32,33].

Nanoscale grain boundaries further resist penetration: as shown in Figure 7b, jagged nanolayers (approximately 0.2 μm thick) at grain boundaries make the paths for low-melting slag phases more twisted. This blocks their spread between grains, forming “nanoscale diffusion barriers.”

For aluminosilicate refractories, mullite formed at high temperatures repairs grain boundary defects through a “self-healing” process, as shown in Figure 7c. Initially (on the left in Figure 7c), small pores (micron-scale) let slag penetrate. When temperatures reach 1400–1600 °C (in the middle in Figure 7c), liquid phases rich in Al and Si at grain boundaries react with CaO in the slag, forming needle-like mullite (0.5–1 μm long). This mullite grows from pore edges toward the center, partially covering the pores. During cooling (on the right in Figure 7c), mullite fully fills the pores, creating a dense “self-healing grain boundary layer” that reduces slag penetration depth by over 40% compared to the initial state [34,35].

In aluminosilicate refractories, the oriented growth of mullite phases results in anisotropic thermal conductivity—the thermal conductivity along the long axis of mullite crystals is 15–20% higher than that along the short axis. This may cause local fluctuations in the temperature gradient at the interface between refractories and the slag layer, affecting slag-layer stability. Such influence can be mitigated by regulating the sintering process (e.g., controlling cooling rates) to reduce the oriented arrangement of mullite phases.

**Figure 8:**
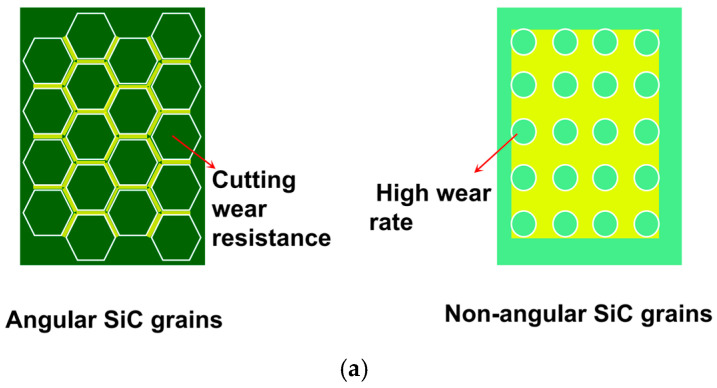

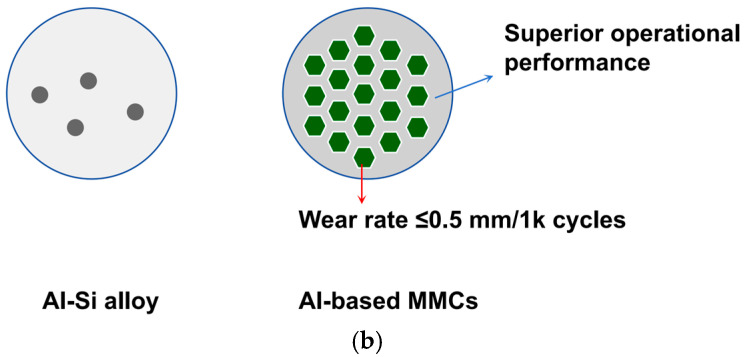
Wear resistance mechanisms: (**a**) structural regulation of silicon-carbide-based materials; and (**b**) application cases of Al-Si alloys and Al-based MMCs.

Wear resistance comes from how well the hard phases and grain boundary materials work together, as shown in Figure 8.

In silicon-carbide-based materials, angular SiC grains (hardness ≥ 28 GPa, on the left in Figure 8a) improve resistance to cutting wear by making grain boundaries harder to shear. Thin glass layers between grains (0.1 μm thick, 5–8% by volume) absorb impact energy by bending slightly, stopping cracks from spreading along boundaries and keeping wear rates below 0.5 mm per thousand heats. In contrast, rounded grains with thicker glass layers (0.3 μm, on the right in Figure 8a) wear much faster because they lack strong hard phase support [36].

Similarly, as shown in Figure 8b, comparisons of Al-Si alloys and aluminum-based composites (MMCs) show that added angular SiC particles (0.2 μm hexagonal shape, on the right in Figure 8b) slow down dislocation movement. This improves properties like high-temperature strength compared to traditional Al-Si alloys (with round Si particles, on the left in Figure 8b) while keeping wear rates low [37].

## 3. Coupling Mechanism Between Slag-Coating Capacity and Self-Protection Capacity

**Figure 9:**
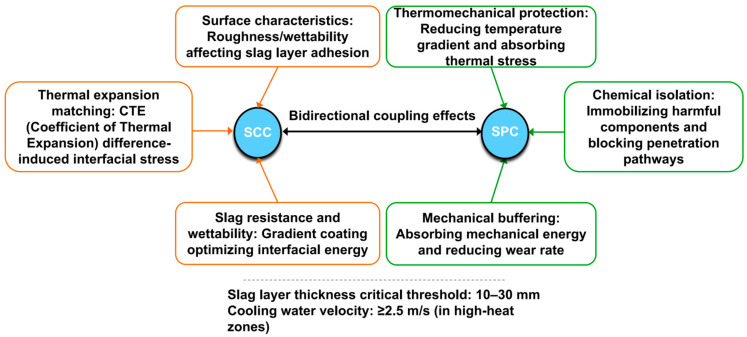
Dynamic coupling flowchart of SCC and SPC.

The slag-coating capacity (SCC) and self-protection capacity (SPC) of blast-furnace staves are core factors ensuring the long-life and efficient operation of blast furnaces. SCC refers to the ability of stave surfaces to form stable slag layers by adsorbing molten slag, which effectively isolates high-temperature hot metal and gas from direct erosion. SPC denotes the damage resistance of stave refractories under combined thermal, chemical, and mechanical stresses. These two capabilities are not isolated but form dynamic coupling through thermodynamic, chemical, and mechanical mechanisms, as illustrated in Figure 9. This coupling mechanism directly influences blast-furnace operational stability, energy consumption, and stave service life. Although the slag coating significantly enhances SPC, excessively thick slag layers may lead to excessive thermal resistance, triggering risks of local overheating or even burn-through in staves. Therefore, slag-layer thickness must be synergistically optimized with parameters such as cooling intensity and refractory thermal conductivity [38].

### 3.1. Interaction Mechanisms Between Slag-Coating Capacity and Self-Protection Capacity

#### 3.1.1. Positive Enhancement of Self-Protection Capacity by Slag Coating

A stable slag coating acts as a dynamic barrier, boosting SPC in three key ways:

(1) Thermal protection: Slag has low thermal conductivity (1–2 W/(m·K)), reducing temperature differences across the stave surface. This slows microcrack growth in refractories caused by heat stress. For example, a 10–30 mm slag layer can cut the thermal shock risk of refractories by 40% [2,4].
(2) Chemical isolation: Slag contains CaO and MgO, which react with harmful gases (e.g., K, Na, ZnO vapors) in the furnace to form high-melting-point compounds (e.g., feldspar). This blocks gas penetration along refractory grain boundaries, reducing “alkali-induced cracking” [1].
(3) Mechanical buffering: The slag layer’s slight plasticity absorbs energy from falling materials or gas impacts, lowering refractory wear rates. In the bosh and belly zones, this can reduce surface wear by 30% compared to uncoated staves [3].

#### 3.1.2. Self-Protection Capacity Regulates Slag-Coating Formation

A stave’s SPC, in turn, affects how well slag coatings form and stay intact:

(1) Surface properties and slag adhesion: Refractory surface roughness (Ra = 10–50 μm) and wettability (contact angle 60–90°) determine how well molten slag sticks. Moderate roughness (e.g., micro-grooves) increases contact area, but excessive roughness can cause stress concentration and slag detachment. For example, Al_2_O_3_-SiC coatings with controlled roughness (Ra = 20 μm) improve slag adhesion strength by 25% [28].
(2) Thermal expansion matching: Refractories and slag must have similar thermal expansion coefficients (CTE) to avoid interface stress. If CTE mismatch exceeds 2 × 10^−6^/°C, cooling can cause slag to crack or peel. SiC-added refractories (CTE 3–4 × 10^−6^/°C) match blast-furnace slag (CTE 4–5 × 10^−6^/°C), doubling slag-layer lifespan [39].
(3) Balancing penetration resistance and wettability: High-Al_2_O_3_ refractories resist slag penetration but have poor wettability (contact angle > 120°). Nano-coatings (e.g., ZrO_2_) solve this by reducing contact angle to 70–80° while blocking FeO penetration, cutting erosion rates by 35% [40].

### 3.2. Realization Pathways of Self-Protection Capacity

SPC is achieved through material design, structural optimization, and process control, working together to resist combined thermal, chemical, and mechanical damage.

#### 3.2.1. Material Design and Property Optimization

(1) Refractory composition: Adding low-expansion phases (e.g., SiC, Si_3_N_4_) adjusts CTE and thermal conductivity to reduce heat stress. For example, SiC-Al_2_O_3_ composites (CTE 4 × 10^−6^/°C) withstand 500 + thermal cycles (20–1200 °C) without cracking. Nano-oxides (ZrO_2_, TiO_2_) refine grain boundaries, reducing slag penetration depth by 30% [25].
(2) Functional coatings: Plasma-sprayed Al_2_O_3_-TiO_2_ coatings (thickness 100–200 μm) lower the contact angle to 60–70°, improving slag adhesion. SiC coatings form a SiO_2_ film at high temperatures, resisting oxidation and alkali corrosion [41].

#### 3.2.2. Interfacial Engineering and Structural Innovation

(1) Gradient structures: Refractory composition changes from surface to interior (e.g., Al_2_O_3_ content 60%→85%), with the surface prioritizing slag adhesion and the interior enhancing erosion resistance. This reduces interface stress by 25% [3]. Notably, the gradient composition also modulates interfacial heat transfer: the metal-refractory interface (e.g., copper-SiC) exhibits an interfacial heat-transfer coefficient $h_{\mathrm{metal}\text{-}\mathrm{refrac}}$ of 1000–3000 W/(m^2^·K), which rises to ~2000 W/(m^2^·K) under 0.5–1 MPa interfacial pressure but drops to 500–1000 W/(m^2^·K) if micro-gaps (e.g., thermal stress cracks) form.
(2) Porosity control: Surface porosity (15–20%) improves slag wetting, while inner porosity (<5%) blocks penetration. This “outer porous, inner dense” structure cuts slag penetration by 40% [33]. The porosity gradient also affects the refractory-slag interface: $h_{\mathrm{refrac}\text{-}\mathrm{slag}}$ ranges 500–1500 W/(m^2^·K), with dense glassy slag (containing mullite) yielding ≥1000 W/(m^2^·K) and porous slag dropping to ≤800 W/(m^2^·K) (measurable via high-temperature laser flash analysis).
(3) Cooling system optimization—zonal cooling strategy: Based on the thermal load distribution in different blast-furnace regions (e.g., high-heat bosh, medium-low heat stack), dynamic regulation of cooling water-flow rate and velocity balances thermal resistance and cooling efficiency [3]. For example, high-flow velocity (≥2.5 m/s) is adopted in high-heat zones to enhance heat dissipation, while low-flow velocity reduces energy consumption in low-heat zones. Staves in different furnace regions require differentiated designs: the hearth uses low-heat-flux copper staves to delay slag skin detachment, whereas the stack employs high-heat-flux cast-iron staves to accelerate slag skin regeneration [1]. Zhang et al. demonstrated in experiments that slag-layer uniformity differences for copper, steel, and iron cooling plates are 2, 5, and 6 mm, respectively. When gas temperature exceeds 1550 °C, steel cooling plates exceed their limiting operating temperature; copper cooling plates with thicknesses of 55–155 mm show almost no change in slag-layer distribution and plate temperature. High-heat-load regions should use copper cooling plates, while low-heat-load regions can adopt iron/steel cooling plates or reduce copper-plate thickness to 20 mm [42].

#### 3.2.3. Process Regulation and Dynamic Adaptation

(1) Slag composition control: Adjusting burden to increase MgO (5–8%) raises slag viscosity (>1.2 Pa·s), reducing fluidity and penetration. Controlling the CaO/SiO_2_ ratio (1.1–1.3) promotes crystallization of high-melting minerals (e.g., merwinite), stabilizing the slag layer [38].
(2) Operational parameter matching: Increasing coal injection requires lowering slag FeO content (<1.5%) to avoid refractory reduction damage. Real-time adjustment of cooling intensity based on top gas analysis keeps slag thickness within 10–30 mm [42].

## 4. Paradigm Innovation of the Fractional Heat-Transfer Model

### 4.1. Advantages of the Fractional Model

Traditional integer-order models rely on assumptions of material homogeneity and instantaneous heat transfer, making it difficult to depict two essential characteristics of the blast-furnace stave system: the cross-scale thermal memory effect (fractal heat flow paths) caused by micro-porosity in the slag layer and grain boundaries in refractories, and the non-local diffusion behavior triggered by thermal relaxation at the metal-refractory interface [43,44,45]. In comparison with traditional models, the fractional heat-transfer model, by introducing non-integer-order derivatives, can quantitatively describe the time lag of heat flow (such as the historical dependence during the slag-layer solidification process) and spatial non-locality (such as the long-range influence of interfacial thermal resistance) [46]. Table 2 systematically compares the core differences between the two types of models in handling heat transfer in the multiphase heterogeneous system of blast furnaces from three aspects: physical mechanism, mathematical form, and engineering applicability [47,48,49,50,51].

However, the fractional heat conduction model still faces practical challenges. First, solving fractional differential equations involves calculating non-integer derivatives, which demands high-performance numerical algorithms. Existing methods (like finite difference or finite element methods) approximate solutions through discretization, which can lead to high computational costs—especially for 3D heat conduction problems [52,53,54]. Second, the physical meaning of fractional-order models is not fully clear. Choosing the right model order and parameters under different material properties and operating conditions requires real-world data, and computing these values for specific engineering scenarios needs more research.

### 4.2. Cross-Disciplinary Inspiration from Existing Fractional Heat-Transfer Models

Although fractional heat-transfer models have not been directly applied in the blast-furnace field, research outcomes from other industrial and natural systems can provide methodological references for modeling blast-furnace staves, as detailed in Table 3 [55,56,57,58,59,60].

### 4.3. Model Construction and Validation Strategy

The variable-order fractional finite element method (VO-FEM) is adopted to discretize the fractional heat-transfer equation into the following:

(1) $$\rho c\frac{\partial^{\alpha }T}{\partial t^{\alpha }}=\nabla \cdot (k\nabla^{\beta }T)+Q_{source}$$

Here, the time- and space-fractional derivatives describe the system’s memory effect and non-local effect, respectively. Where 0<α≤1, 0<β≤1; ρ denotes the material density, $\mathrm{kg}/m^{3}$; c represents the specific heat capacity; and $J\cdot kg^{-1}\cdot {{}^{\circ}C}^{-1}$; $Q_{source}$ is the heat source term.

In practical stave problems, two common types of boundary conditions are typically encountered: convective boundary conditions and adiabatic boundary conditions. They respectively reflect heat exchange between the stave and the external environment (e.g., cooling fluids). The mathematical expressions for convective boundary conditions are as follows:

Interface between furnace wall and air:

(2) $$-k\nabla^{\beta }T\cdot \hat{n}=h_{\mathrm{air}}(T-T_{\mathrm{air}})$$

Interface between water and water pipe wall:

(3) $$-k\nabla^{\beta }T\cdot \hat{n}=h_{\mathrm{water}}(T_{\mathrm{pipe}}-T_{\mathrm{water}})$$

Interface between inner surface and gas:

(4) $$-k\nabla^{\beta }T\cdot \hat{n}=h_{\mathrm{gas}}(T_{\mathrm{inside}\text{-}\mathrm{wall}}-T_{\mathrm{gas}})$$

The adiabatic boundary condition:

(5) $$-k\nabla^{\beta }T\cdot \hat{n}=0$$

where $T_{\mathrm{air}}$ is the air temperature; $T_{\mathrm{pipe}}$ is the water pipe wall temperature; $T_{\mathrm{water}}$ is the water temperature; $T_{\mathrm{inside}\text{-}\mathrm{wall}}$ is the inner surface temperature of the stave; $T_{\mathrm{gas}}$ is the gas temperature; and $\hat{n}$ is the normal unit vector, pointing to the external fluid.

Notably, interfacial heat-transfer coefficients are critical input parameters: $h_{\mathrm{metal}\text{-}\mathrm{refrac}}$ is set to 1000–3000 W/(m^2^·K), varying with interfacial pressure (e.g., ~2000 W/(m^2^·K) at 0.5–1 MPa) and micro-gap presence (dropping to 500–1000 W/(m^2^·K) if cracked); and $h_{\mathrm{refrac}\text{-}\mathrm{slag}}$ ranges 500–1500 W/(m^2^·K), depending on slag compactness (≥1000 W/(m^2^·K) for dense glassy slag, ≤800 W/(m^2^·K) for porous slag), measurable via high-temperature laser flash method. Sensitivity analysis confirms $h_{\mathrm{refrac}\text{-}\mathrm{slag}}$ fluctuations affect slag temperature distribution by ±15%, requiring experimental calibration.

To solve the 3D fractional heat-transfer model numerically, we modify traditional methods like finite difference or finite element analysis to handle fractional derivatives [61,62,63,64,65,66]. Sensitivity analysis (testing how changes in cooling water velocity, stave thickness, slag-layer thickness, or thermal conductivity affect results) helps optimize stave design, improving cooling efficiency and self-protection.

To validate the accuracy and application potential of the 3D fractional heat-transfer model, it is recommended to conduct numerical simulation and experimental comparison through the following approaches:

(1) Experimental Design: Representative blast-furnace stave regions (e.g., top, bosh, and hearth) should be selected as test objects during experimentation, with emphasis on structural designs and refractory parameters (e.g., thermal conductivity, heat capacity) that significantly influence heat-transfer processes. Control groups must be established for different design schemes (e.g., stave thickness, cooling tube arrangements), and temperature data, heat-flux density, and thermal states should be collected under diverse operating conditions.
(2) Experimental Data Acquisition: High-precision temperature sensors and heat-flux meters are employed to collect data during actual furnace operations. Critical measurements—such as temperature differences between stave surfaces and cooling tubes and heat-flux density distributions—provide essential real-world data for numerical simulations. Tests should be conducted across different furnace types, stave designs, and refractory materials to comprehensively evaluate the model’s performance.
(3) Numerical Simulation Methodology: A 3D fractional heat-transfer model is established to replicate experimental conditions, accounting for heterogeneous material properties, complex geometries, and fluctuating in-furnace heat loads. Specifically, when slag coating affects the stave, appropriate thermal resistance models should be incorporated to accurately reflect the inhibitory effect of slag on heat transfer.
(4) Comparative Analysis: Experimental data and simulation results are compared to assess the model’s performance in predicting stave temperature distribution, heat-flux density, and heat load transfer. Key metrics include accuracy of temperature fields, reasonableness of heat-flux density distributions, and the model’s capability to capture nonlinear heat conduction characteristics. Significant discrepancies between model predictions and experimental results should trigger cause analysis and further model optimization.

**Figure 10:**
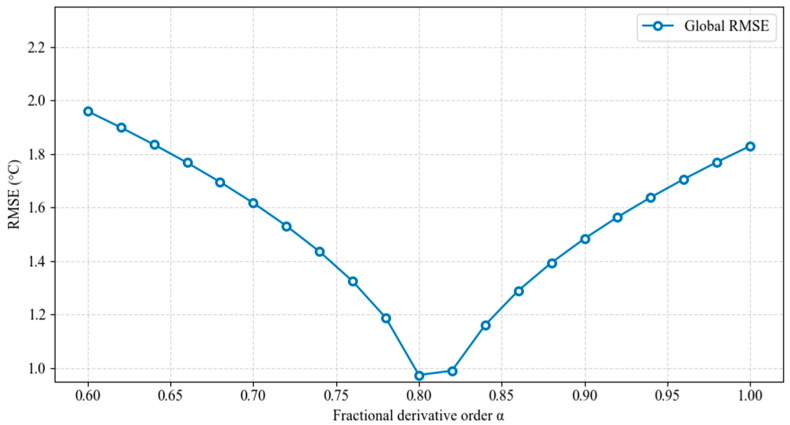
Global error-order relationship diagram.

Based on the aforementioned methodology, the research team validated the effectiveness of the fractional-order model through preliminary industrial trials (on-site testing in the belly zone of a 5500 m^3^ blast furnace). This verification aims to demonstrate the model’s potential advantages over conventional approaches through theoretical derivation and empirical comparison, thereby providing references for subsequent studies. As shown in Figure 10, the derivative order α of the heat-transfer equation varied within the range [0.6, 1]. The fractional-order model (α=0.8) achieved a root mean square error (RMSE = 0.99 °C) for global temperature prediction, representing a 50% reduction compared to the integer-order model (α=1, RMSE = 1.98 °C), which verifies the unique advantage of fractional calculus in characterizing historical heat accumulation effects during unsteady heat-transfer processes. The V-shaped distribution of RMSE demonstrates that the fractional-order model, through optimized thermal memory weighting, not only overcomes the integer-order model’s underestimation of transient thermal shocks but also avoids steady-state deviations caused by excessive memory effects.

The limitations of traditional integer-order models in blast-furnace slag-layer thickness prediction have been substantiated through both theoretical research and industrial measurements. In refractory-slag interface studies, Liu et al. demonstrated that when porosity exceeds 15%, conventional models neglecting fractal pore structures (fractal dimension: 1.8–2.2) exhibit temperature gradient prediction errors of ±18–22% [6]. This discrepancy arises from the oversimplified assumption of linear heat conduction paths, whereas actual porous media require heat flow to navigate complex pore networks, reducing equivalent thermal conductivity by over 30%.

Industrial validation in a 2580 m^3^ blast-furnace belly zone further confirmed these findings. Liu et al. revealed that traditional integer-order models failed to capture the dynamic spalling-regeneration process of slag layers (with 0.5–3 h time lags), resulting in ±6 mm prediction errors (±20% deviation) for 10–30 mm thick slag layers [18]. The fundamental cause was identified as the oversimplified treatment of fractal heat transfer in micro-pores.

Cross-disciplinary simulations reinforced this universal limitation. Tang et al.’s cyclone slag-layer modeling showed that disregarding pore fractal dimension led to solidification time prediction errors of ±20–25% [7]. Collectively, these results demonstrate that traditional models’ inherent constraints in complex multiphase media stem from their inability to incorporate thermal memory effects and non-local diffusion phenomena.

## 5. Future Research Directions and Challenges

**Figure 11:**
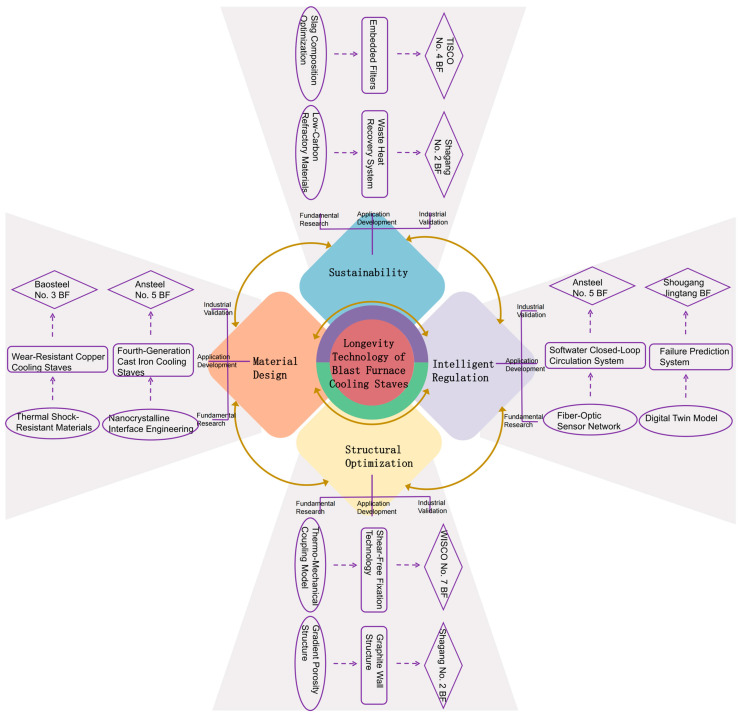
Long-life technology roadmap for blast-furnace staves [69,70,71,72,73,74,75,76,77,78,79,80,81,82,83].

The study of slag-coating mechanisms in blast-furnace staves still faces critical scientific issues and engineering challenges, requiring breakthroughs through multidisciplinary research. Guided by the technical roadmap in Figure 11, specific directions for four research dimensions are outlined below.

### 5.1. Multiscale Modeling and Advanced Simulation Techniques

Synergistic analysis of microstructures and macro-properties offers a new paradigm for understanding dynamic slag-coating mechanisms. A multiscale computational framework integrating molecular dynamics (atomic-scale interactions), phase-field models (interface evolution), and finite element analysis (thermo-mechanical coupling) can reveal how material heterogeneities (e.g., grain boundaries, porosity) regulate slag wetting behavior and spalling resistance. Such models must be validated experimentally using high-temperature in situ synchrotron imaging or laser confocal microscopy to capture real-time slag-refractory interfacial reactions [67,68].

### 5.2. Development of Next-Generation Refractory Materials

Current copper-based/cast-iron staves exhibit limited durability under extreme thermal cycling (>1300 °C) and chemical corrosion (alkali metal/zinc erosion). Research directions for new material systems include the following:

(1) Oxide–carbide gradient composites: mitigating interfacial stress from thermal shock through tailored coefficients of thermal expansion (CTE).
(2) High-entropy alloy coatings: FeCoNiCrAl layers prepared via cold spraying to enhance erosion resistance while maintaining thermal conductivity.
(3) Self-healing ceramics: incorporating microencapsulated metallic phases (e.g., Si) that oxidize to form protective SiO_2_ layers upon slag penetration.

### 5.3. Intelligent Monitoring and Adaptive Control Systems

Machine-learning algorithms demonstrate significant potential in enhancing the prediction accuracy and efficiency of blast-furnace temperature and slag deposition behavior, effectively addressing the limitations of traditional mechanistic models. Research indicates that machine-learning models can precisely capture complex nonlinear relationships between operational parameters (e.g., cooling water-flow rate, furnace temperature, slag composition) and slag-layer thickness. For instance, a long short-term memory (LSTM) network trained on 10-year operational data from five blast furnaces (covering belly, bosh, and lower shaft zones) using 12 key parameters (including heat-flux density, refractory temperature gradient, and pulverized coal injection rate) achieved 92% prediction accuracy for slag-layer thickness—a 17% improvement over traditional integer-order models (75%). This advantage stems from its ability to learn implicit temporal correlations, such as the 0.5–3 h lag effect between cooling intensity adjustments and slag-layer regeneration—relationships difficult to quantify in mechanistic models.

Physics-informed neural networks (PINNs), an emerging framework integrating physical mechanisms with data-driven approaches, exhibit unique advantages in predicting blast-furnace slag behavior. Unlike purely data-driven models, PINNs enforce strict physical constraints by embedding heat conduction equations (e.g., fractional-order Caputo derivative models) as regularization terms in the loss function, making them particularly suitable for high-temperature industrial scenarios with limited data. The coupling of PINNs with fractional-order models further highlights synergistic value: incorporating non-local heat conduction characteristics described by Caputo derivatives as physical constraints reduces reliance on industrial data volume (achieving traditional model accuracy with only 500 samples versus 1000) while addressing the high computational complexity of mechanistic models. This “physical mechanism + data learning” fusion paradigm provides an efficient solution for real-time iteration in blast-furnace digital twin systems.

The integration of IoT sensor networks (e.g., fiber Bragg grating thermocouples, acoustic emission detectors) with machine-learning algorithms enables predictive maintenance. Key technical frontiers include

(1) Digital twin platforms: coupling real-time thermal data with mechanistic models to predict slag-layer thickness evolution and optimize cooling parameters.
(2) Closed-loop control systems: precise regulation of water-flow velocity (±0.1 m/s accuracy) based on slag-layer thermal resistance feedback to prevent local overheating.

### 5.4. Environmental Sustainability and Circular Economy

Carbon neutrality goals impose new requirements for the full lifecycle management of staves:

(1) Low-carbon material synthesis: developing microwave-sintered refractories to reduce energy consumption by 30–50% compared to traditional sintering processes.
(2) High-value utilization of furnace slag: designing stave surfaces that generate CaO-Al_2_O_3_-SiO_2_ slag layers directly applicable for cement production.
(3) Resource recovery systems: embedded porous filters in staves to capture and recycle Zn/alkali metal vapors, reducing toxic emissions by over 90%.

## 6. Conclusions and Prospects

Research on slag-coating mechanisms in blast-furnace staves has revealed the critical role of material design and process optimization in enhancing equipment performance. Current copper–steel composite plate components, through welded water channel covers and convex structure design, significantly enhance the sealing performance and heat conduction efficiency of cooling water channels. Meanwhile, the combined application of castables and anchors effectively reduces the manufacturing costs of refractories. Studies have confirmed that copper-based staves, leveraging their high thermal conductivity and corrosion resistance, exhibit superior durability to cast-iron materials under extreme operating conditions. However, interfacial stress issues induced by long-term thermal cycling still require resolution through innovative solutions such as gradient composite materials or high-entropy alloy coatings.

Breakthroughs in intelligent monitoring technologies have provided a new paradigm for blast-furnace operation and maintenance. IoT networks based on fiber Bragg grating thermocouples and acoustic emission detectors, combined with digital twin platforms, enable real-time prediction of slag-layer thickness and dynamic optimization of cooling parameters. This integrated system can improve water-flow velocity control precision to ±0.1 m/s, significantly reducing the risk of local overheating. In terms of sustainability, microwave sintering processes reduce refractory energy consumption by 30–50%, while embedded porous filtration systems achieve over 90% zinc vapor recovery, driving the transformation of steel production toward a circular economy model.

Future research should focus on three key directions:

(1) Multiscale interface regulation: combining synchrotron imaging techniques to resolve atomic migration patterns at copper/steel interfaces under thermal stress.
(2) Adaptive material systems: developing temperature-responsive self-healing ceramics that actively form protective oxide layers during slag erosion.
(3) Full lifecycle management: establishing a carbon footprint assessment system covering material synthesis, online monitoring, and slag reuse, aiming to reduce the carbon emission intensity of stave systems by 20% by 2030.

## Figures and Tables

**Table 1:** Design requirements for staves in different regions.

Region	Thermal Load (kW/m^2^)	Erosion Factors	Material Requirements	Cooling Methods
Bosh	300–600	High-temperature thermal shock, alkali metals	Copper substrate, silicon-carbide-based refractories	Forced circulation water cooling
Belly	200–400	Slag erosion, thermal fatigue	Composite structure of cast-iron + copper cooling plates	Mixed water and air cooling
Middle-lower stack	150–250	Slag adhesion, thermal stress	Ductile iron with refractory linings + silicon-carbide coatings	Medium-pressure water cooling

**Table 2:** Comparison of model characteristics.

Comparison Dimension	Traditional Integer-Order Model	Fractional-Order Model (Caputo Derivative)
Physical Assumptions	Homogeneous materials, local instantaneous heat transfer	Heterogeneous multiphase systems, thermal memory/non-local effects
Applicable Scenarios	Simple structures, steady-state heat conduction	Multiscale pores/cracks, transient heat flow
Mathematical Form	Second-order PDE (Fourier’s law)	α order time derivative + β order spatial derivative (α, β ∈ (0, 1))
Engineering Challenges	Fails to capture thermal relaxation and fractal heat flow	High computational complexity

**Table 3:** Application cases of fractional-order heat-transfer models across disciplinary fields.

Application Field	Fractional-Order Model Type	Core Mechanism	BF Cooling Wall Application Focus	Reference
Composite cylinder heat transfer	Time-fractional Caputo model	Non-local heat flux under interfacial thermal contact	Thermal resistance matching analysis of refractory-metal composite structures	[56]
Porous media heat conduction	Multi-interaction continuum model	Fractal heat flow paths influenced by fracture permeability	Modeling thermal memory effect in slag-layer micro-porous structures	[59]
Biological tissue thermal damage	Fractional-order bioheat-transfer model	Thermal damage threshold calculation under transient heat-flux disturbances	Simulation of sudden temperature change on cooling wall surface due to slag crust detachment	[60]

## Data Availability

No new data were created or analyzed in this study. Data sharing is not applicable to this article.
